# Overexpression of centromere protein K (CENPK) in ovarian cancer is correlated with poor patient survival and associated with predictive and prognostic relevance

**DOI:** 10.7717/peerj.1386

**Published:** 2015-11-05

**Authors:** Yi-Chao Lee, Chi-Chen Huang, Ding-Yen Lin, Wen-Chang Chang, Kuen-Haur Lee

**Affiliations:** 1 Graduate Institute of Neural Regenerative Medicine, College of Medical Science and Technology, Taipei Medical University, Taipei, Taiwan; 2 Institute of Bioinformatics and Biosignal Transduction, College of Bioscience and Biotechnology, National Cheng Kung University, Tainan, Taiwan; 3 Graduate Institute of Cancer Biology and Drug Discovery, College of Medical Science and Technology, Taipei Medical University, Taipei, Taiwan; 4 Graduate Institute of Medical Sciences, College of Medicine, Taipei Medical University, Taipei, Taiwan

**Keywords:** Ovarian cancer, CENPK, CA125, HE4

## Abstract

Ovarian cancer has a poor prognosis. Most patients are diagnosed with ovarian cancer when the disease has reached an advanced stage and cure rates are generally under 30%. Hence, early diagnosis of ovarian cancer is the best means to control the disease in the long term and abate mortality. So far, cancer antigen 125 (CA125) and human epididymis protein 4 (HE4) are the gold-standard tumor markers for ovarian cancer; however, these two markers can be elevated in a number of conditions unrelated to ovarian cancer, resulting in decreased specifically and positive predictive value. Therefore, it is urgent to identify novel biomarkers with high reliability and sensitivity for ovarian cancer. In this study for the first time, we identified a member of the centromere protein (CENP) family, CENPK, which was specifically upregulated in ovarian cancer tissues and cell lines and the overexpression of which was associated with poor prognoses in patients with ovarian cancer. In addition, the presence of CENPK significantly improved the sensitivity of CA125 or HE4 for predicting clinical outcomes of ovarian cancer patients. In conclusion, we identified that CENPK was specifically upregulated in ovarian cancer cells and can be used as a novel tumor marker of ovarian cancer.

## Introduction

Ovarian cancer is the most lethal gynecologic malignancy in women, with 21,290 estimated new cases and 14,180 estimated deaths in 2014 in the US alone (Siegel, Miller & Jemal, 2015). In the early stages of ovarian cancer, no symptoms are evident, or symptoms are similar to other benign gynecological diseases (Bast, Hennessy & Mills, 2009). Thus, most of these tumors are detected at an advanced stage (particularly in stage III) with metastases present beyond the ovaries precluding curative treatment (Modugno, Ovarian & High-Risk Women Symposium P, 2003). However, differences in 5-year survival among patients with tumors in stage III are noticeable, ranging from 59% for patients with stage IIIa tumors to 40% and 29% for patients with stage IIIb and stage IIIc tumors, respectively (Heintz et al., 2001). Hence, identification and validation of specific novel biomarker for diagnosing ovarian cancer is the best means to control the disease in the long term and abate mortality (Rauh-Hain et al., 2011).

The centromere is the default chromosomal region onto which the mitotic/meiotic kinetochore gradually assembles to ensure correct chromosome attachment to microtubules and equal segregation of sister chromatids (Perpelescu & Fukagawa, 2011). The kinetochore is the protein structure on chromatids where spindle fibers attach during cell division to pull sister chromatids apart (Gassmann et al., 2012). Moreover, kinetochores consist of more than 16 different proteins. Many of these proteins which help the kinetochore associate with DNA are conserved among eukaryotic species (Perpelescu & Fukagawa, 2011). Centromere protein A (CENPA) was one of the first identified kinetochore components in humans. It is a unique histone H3-like protein only found in active centromeres and is involved in the epigenetic maintenance of centromere identity (Black et al., 2007). *Cenpa* (symbol for mouse *CENPA*) gene knockout and *hcp-3* (*CENPA* homologue in *Caenorhabditis elegans*) gene suppression results in severe mitotic segregation problems and early embryonic death (Buchwitz et al., 1999; Howman et al., 2000). These results suggest that CENPA plays an important role in cell cycle regulation and cell survival. Recently, the overexpression of CENPA was also identified in several human malignancies, including hepatocellular carcinoma (Li et al., 2011), colorectal cancer (Tomonaga et al., 2003), lung adenocarcinoma (Wu et al., 2012), breast cancer (McGovern et al., 2012), and ovarian cancer (Qiu et al., 2013). The prognostic significance of CENPA was described for various cancers. For instance, Qiu et al. (2013) reported that CENPA is upregulated in epithelial ovarian cancer and predicts poor outcomes in patients with this disease. CENPA also shows a poor prognostic impact in estrogen receptor-positive breast cancer (McGovern et al., 2012). Taken together, these data suggest that CENPA might serve as a tumor marker in cancers.

In addition to CENPA chromatin, human centromeres contain at least 16 nonhistone proteins distributed in several functional groups as follows: CENPC, CENPH/CENPI/CENPK, CENPL/CENPM/CENPN, CENPO/CENPP/CENPQ/CENPR/ CENPU, CENPT/CENPW, and CENPS/CENPX (Amano et al., 2009; Hori et al., 2008; Izuta et al., 2006; Okada et al., 2006). One role of CENP family proteins is to recruit outer kinetochore components, such as KNL1, the Mis12 complex, and the Ndc80 complex (KMN network), onto which spindle microtubules attach with their structural and regulatory proteins (Perpelescu & Fukagawa, 2011). Two other centromere proteins, CENPE and CENPF, are localized in the fibrous corona from the *G*_2_/*M* phase onwards and travel to the mid-zone together with proteins that act in the spindle checkpoint (Rattner et al., 1993; Yen et al., 1991). Among the above-mentioned CENP family proteins, CENPE, CENPF, CENPH, and CENPJ have significant positive hits in the Catalogue of Somatic Mutations in Cancer database for cancer-associated mutations (Bamford et al., 2004). However, correlations between expression levels of these CENP family proteins and cancers remain largely unclear.

## Materials and Methods

### Cell culture

The human ovarian papillary serous cystoadenocarcinoma cell line, OC314, was obtained from the ICLC Animal Cell Lines Database (Servizio Biotecnologie IST, Centro di Biotecnologie, Avanzate L.go R. Benzi, Genova, Italy). Cells were propagated in RPMI 1640 medium (Life Technologies, Rockville, MD, USA) supplemented with 5% fetal bovine serum (FBS; Life Technologies) and 2 mM L-glutamine (Sigma-Aldrich, St. Louis, MO, USA). Other human cell lines including TOV-112D (derived from an ovarian endometrioid carcinoma), TOV-21G (derived from an ovarian clear cell carcinoma), H184B5H5/M10 (human mammary epithelial cells), T/G HA-VSMC (human normal aorta smooth muscle cells), and HFL1 (lung fibroblasts) were obtained from the Bioresources Collection and Research Center (BCRC, Hsinchu, Taiwan). TOV-112D and TOV-21G cells were propagated in a 1:1 mixed medium of MCDB 105 (Sigma-Aldrich) and Medium 199 (Life Technologies) supplemented with 15% FBS. H184B5H5/M10 cells were propagated in GIBCO 11900 medium (Life Technologies) supplemented with 10% calf serum (Life Technologies). HFL1 and T/G HA-VSMC cell lines were propagated in Ham’s F12K medium (HyClone, Logan, UT, USA) supplemented with 10% FBS.

### Digital gene-expression displayer

The electronic profiling of differentially expressed of gene expression levels of CENP family, including CENPA, CENPE, CENPF, CENPJ, CENPH/I/K group: CENPH and CENPK, CENPL/M/N group: CENPL, CENPOP/Q/R/U group: CENPQ and CENPT/W group: CENPT in various human cancers was used online bioinformatic tool freely available from the National Cancer Institute Cancer Genome Anatomy Project (CGAP) gene expression database (http://www.ncbi.nlm.nih.gov/ncicgap/) (Mitelman, Mertens & Johansson, 1997). The gene expression levels of CENP family in various human cancers was analyzed by using expressed sequence tag (EST) probe from complementary DNA (cDNA) expression library (http://cgap.nci.nih.gov/Tissues/GXS).

### Reverse transcription (RT) and quantitative polymerase chain reaction (PCR) assays

Total RNA was extracted using the Trizol reagent (Life Technologies) following the manufacturer’s recommendations. Purified RNA was treated with RNase-free DNase I (Ambion, Austin, TX, USA) to remove residual genomic DNA contamination following the manufacturer’s protocol. Complementary (c)DNA synthesis and a quantitative real-time RT-PCR was performed using the TITANIUM One-Step RT-PCR kit (Clontech, Palo Alto, CA, USA) containing SYBR Green I (BioWhittaker Molecular Applications (BMA), Rockland, ME, USA). The RT-PCR mixtures were incubated at 50 °C for 1 h and 95 °C for 10 min, and then 40 PCR cycles were conducted (95 °C for 30 s, 65 °C for 30 s, and 68 °C for 60 s). Sequences of primers included: 5-GAAACACTCACCGATTCAAATG-3 and 5-GCTTTT-GGAACTCTTCTTTTCC-3 for CENPK; and 5-CTGGACTTCGAGCAAGAGATG-3 and 5-TGATGGAGTTGAAGGTAGTTTCG-3 for *β*-actin. Real-time fluorescence monitoring and a melting-curve analysis were performed with Rotor-Gene 3000™ and Rotor-Gene 3000 operating software vers. 4.6.94 (Corbett Research, Sydney, Australia). Negative controls containing no cDNA template were included in each experiment. A melting curve was created at the end of the PCR cycle to confirm that a single product had been amplified. The relative transcript amount of the target gene, calculated using standard curves of serial cDNA dilutions, was normalized to that of *β*-actin of the same cDNA.

### Cancer profiling array assay

Cancer Profiling Array II (Clontech, Palo Alto, CA, USA) which includes ubiquitin-normalized cDNA from 154 tumor and corresponding normal tissues from individual patients was used to discriminate specific gene expression profiles among different cancer types. Using rediprime™ II (Amersham Biosciences, Buckinghamshire, UK) and *α*-P^32^-dCTP, a full-length CENPK cDNA fragment was labeled and used as a probe to detect CENPK expression in this array.

### RNA Interference (RNAi)

A small interfering (si)RNA oligonucleotide (5-AACACTCACCGATTCAAATGC-3) was designed to target the CENPK sequence. The target sequence (5-AATTCTCCGAACGTGTCACGT-3) which has 16 bases that overlap with *Thermotoga maritimia* (GenBank accession no.: AE001709) section 21 of 136 of the complete genome was used as the negative control siRNA. siRNAs were synthesized with the silencer™ siRNA Construction Kit (Ambion) following the manufacturer’s protocol. siRNA transfection was performed in 24-well plates using Oligofectamine™ (Invitrogen).

### Cell Viability Assay

Cell viability was determined by adding MTT (Sigma-Aldrich) to cell cultures at a final concentration of 0.5 mg/ml. After 2 ∼ 5 h of incubation at 37 °C, dark crystals that had formed were dissolved in DMSO, and the amount was obtained by measuring the absorbance of the solution at 570 nm.

### Genes expression and survival analysis

Genes expression of biochemical marker of epithelial ovarian cancer were analyzed in each group using the SurvExpress web-based tool to provide survival analysis and risk assessment using a biomarker gene list as input to a Cox proportional-hazards regression. Cox regression relates the time of death to a number of explanatory variables known as covariates, in this case genes (Aguirre-Gamboa et al., 2013). A population of ovarian cancer patients (GSE18520) (Mok et al., 2009) were classified in high-risk and low-risk groups for patients of genetic profiles on the basis of survival.

### Statistical analysis

Statistical analyses were performed as recommended by an independent statistician. These included unpaired Student’s *t*-test. All statistical analyses were performed using SPSS software (SPSS, Chicago, IL, USA), all values are expressed as mean ± standard error, and statistical significance was accepted at *p* < 0.05.

## Results

### CENPK was overexpressed in various human cancers

**Figure 1:**
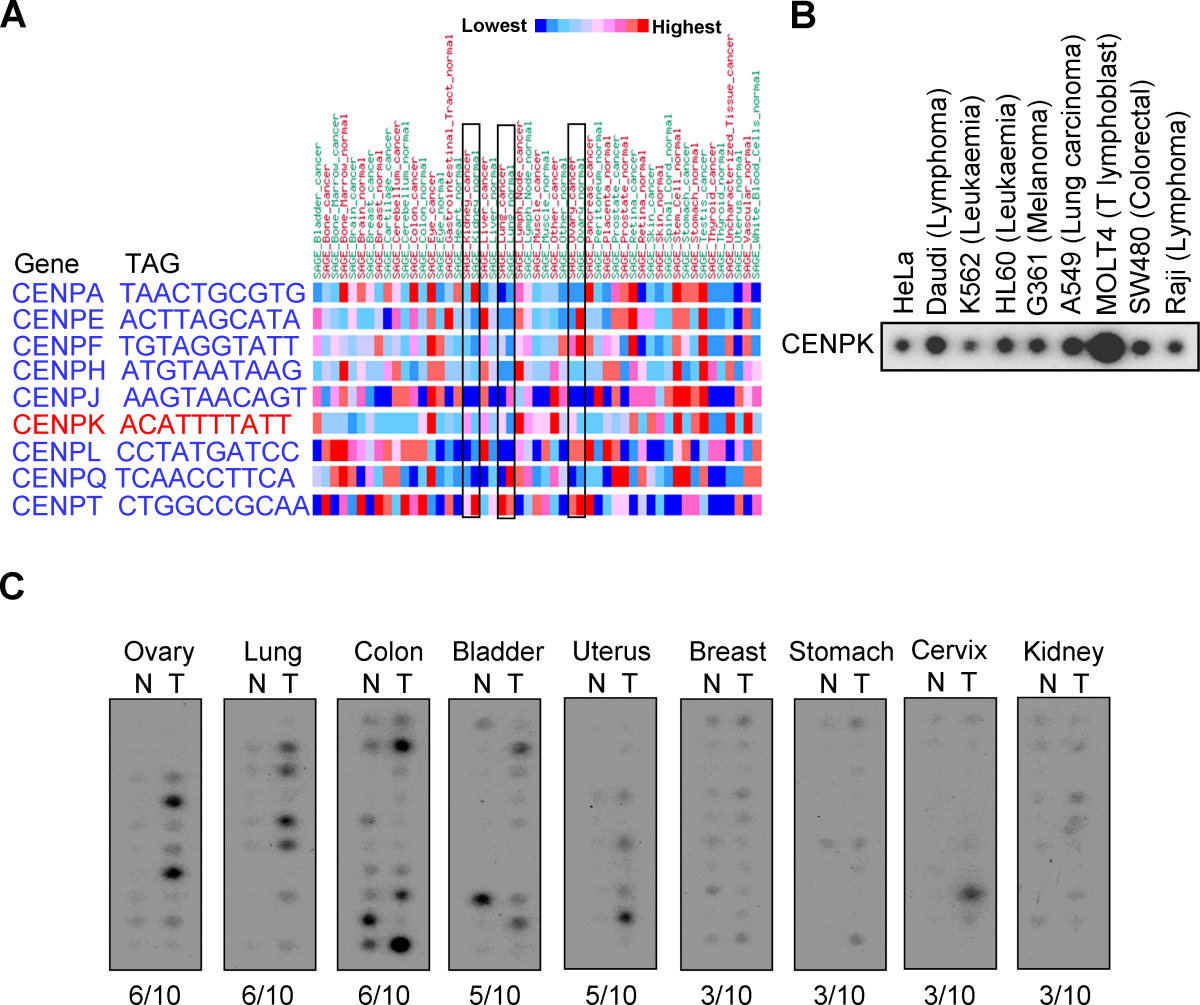
Centrosome protein K (CENPK) was overexpressed in various human cancers. (A) Gene expression levels of CENP family proteins in various human cancers. Expression profiles of human CENPK in cancer cell lines (B) and various tumors (T) and normal (N) tissues (C).

To understand gene expression levels of CENP family in various human cancers, the mRNA expression of levels of CENP family were analyzed by using CGAP gene expression database. Results are shown in Fig. 1A, indicating that among nine CENP family proteins, CENPK was specifically upregulated in kidney, lung, and ovary tumor tissues. To further confirm the expression of CENPK in various cancers, the Cancer Profiling Array II was used to systematically examine messenger (m)RNA expression levels of CENPK in different cancer specimens. Figure 1B shows that CENPK mRNA expression was observed in a majority of cancer cell lines, and was highly expressed in MOLT4, A549, and Daudi cells. In addition, the overexpression of CENPK in cancer specimens with a high average occurrence (>50%) was observed in the tissues such as the ovaries, lungs, and colon (Fig. 1C).

### Knockdown of CENPK expression in ovarian cancer cells causes substantial inhibition of tumor cell growth

**Figure 2:**
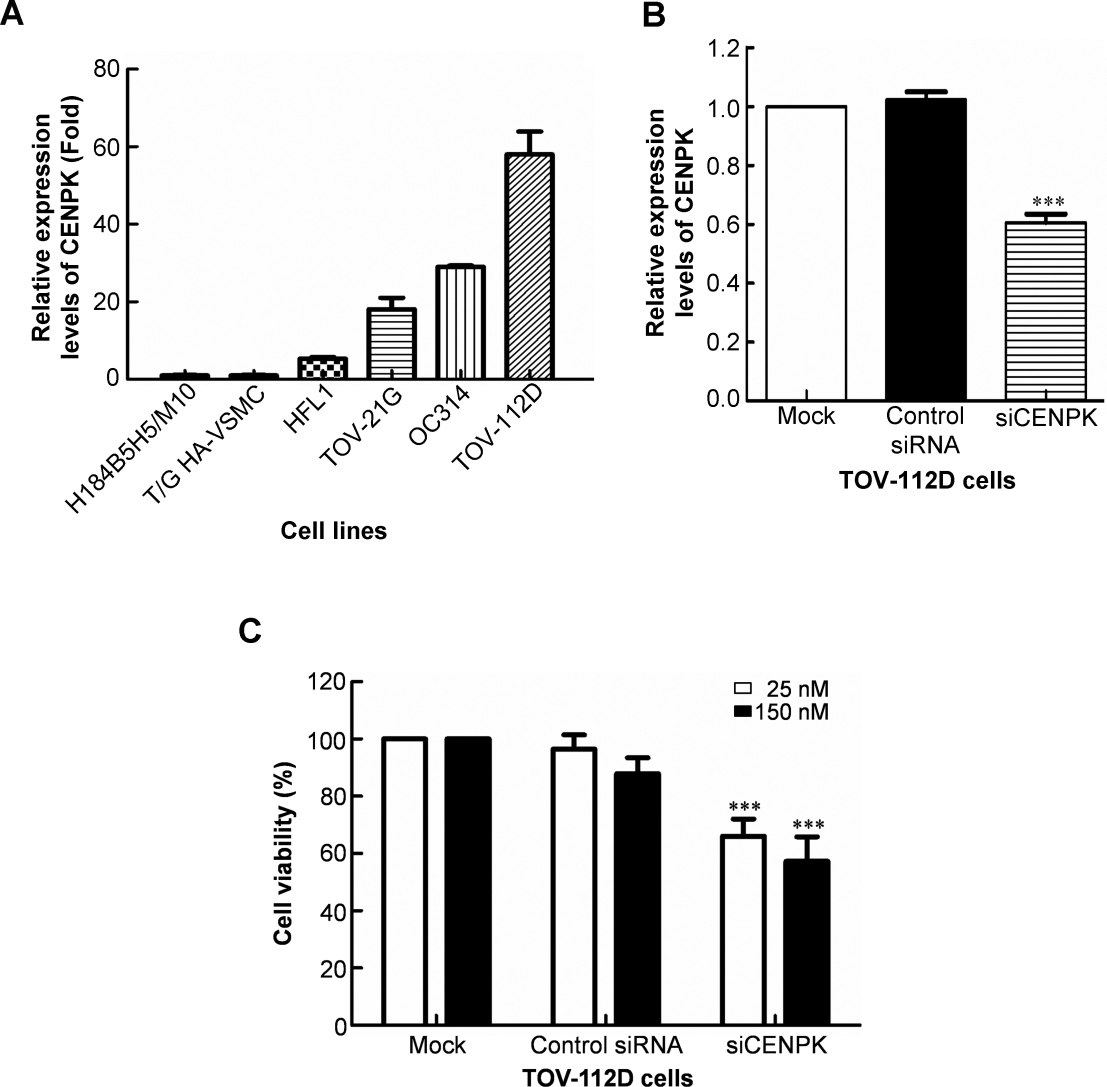
Centrosome protein K (CENPK)-specific siRNA inhibits cell proliferation of ovarian cancer cells. (A) Quantitative RT-PCR analysis of CENPK expression levels in three non-tumorigenic cell lines (H184B5H5/M10, T/G HA-VSMC, and HFL1) and three ovarian cancer cell lines (TOV-21G, OC314, and TOV-112D). (B) Gene-specific siRNA was transiently transfected into TOV-112D cells. (C) Cell proliferation was determined 48 h after transfection by an MTT assay. Values are presented as the mean ± SEM of three experiments from at least two independent siRNA preparations. ^∗∗∗^*p* < 0.001.

The above results implied that CENPK may play a role in cancer development. To understand the roles of CENPK in ovarian cancer, we first analyzed expression levels of CENPK mRNA in three normal cell lines and three ovarian cancer cell lines by a quantitative RT-PCR. Compared to three non-tumorigenic cell lines (H184B5H5/M10, T/G HA-VSMC, and HFL1), CENPK mRNA was highly expressed in ovarian cancer cell lines, including TOV-21G, OC314, and TOV-112D (Fig. 2A). Next, we selected TOV-112D cells as a cell model which displayed the highest endogenous CENPK expression to investigate the roles of CENPK in controlling cellular proliferation. First, we designed and synthesized CENPK siRNA sequences, and the knockdown efficiencies of CENPK siRNA in TOV-112D cells were then evaluated using a quantitative RT-PCR. As shown in Fig. 2B, cells transfected with CENPK siRNA showed significantly reduced (by about 40%) transcription of CENPK mRNA compared to control siRNA and to cells without transfectants. A further examination used MTT to observe the effect of decreasing CENPK levels. As shown in Fig. 2C, 25 or 150 nM of CENPK siRNA transfected into TOV-112D cells caused significantly decreased cell viability at 48 h after transfection. Taken together, these results indicate that CENPK plays an oncogenic role, and RNAi directed against CENPK significantly decreased the growth rate of ovarian cancer cells.

### Ectopic activation of CENPK in ovarian tumors is a strong predictor of a poor prognosis

**Figure 3:**
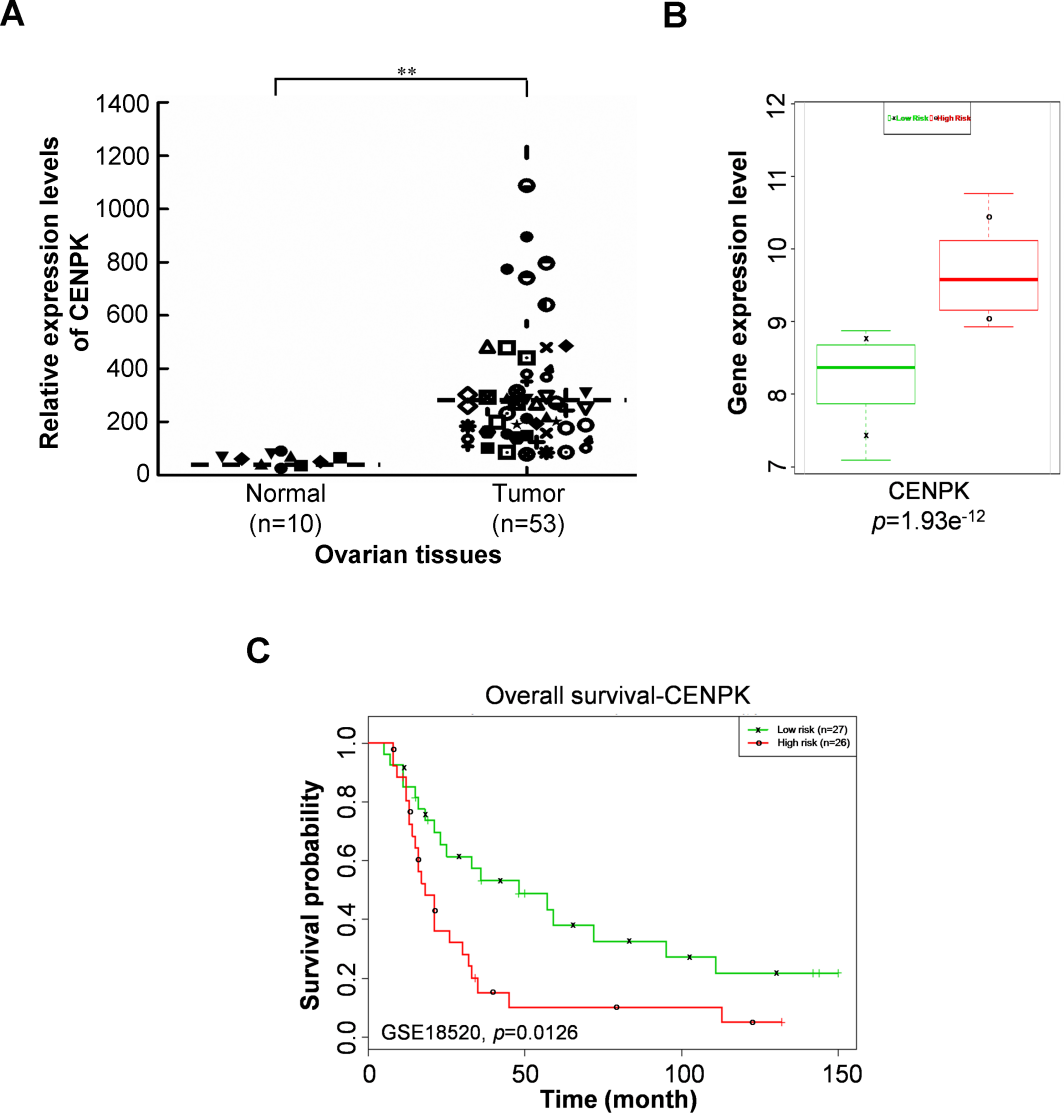
Aberrant expressions of centrosome protein K (CENPK) are associated with shorter survival of ovarian cancer patients. (A) Relative expression levels of CENPK in ovarian tissues analyzed using the public GEO database. (B) Box plots of CENPK gene expressions between risk groups using a *t*-test. (C) Accurate predictions of patient outcomes using Kaplan–Meier analyses of patients with high expression levels of CENPK (high risk) (*n* = 26) showing stratification of CENPK for shorter- versus longer-surviving patients. ^∗∗^*p* < 0.01.

To understand whether the CENPK expression level was associated with clinical outcomes of ovarian tumors, we first investigated CENPK expression in ovarian tissues. We recruited a cohort of 53 ovarian cancer patients from a publicly available dataset, deposited in the NCBI Gene Expression Omnibus (GEO) under accession no. GSE18520 (Mok et al., 2009). As shown in Fig. 3A, we observed that CENPK expression was significantly upregulated in ovarian cancer tissues compared to a normal group. Next, we analyzed the prognostic relevance of CENPK in ovarian cancer using a Kaplan–Meier survival analysis (Aguirre-Gamboa et al., 2013). We analyzed CENPK gene expression level with respect to ovarian cancer in 53 subjects with stage III/IV grade 3 serous ovarian carcinoma, and survival analysis was censored by survival months. Risk analysis was performed in which a predicted risk for a specific patient genetic profile was determined. The subjects were then partitioned into low risk and high risk groups (Aguirre-Gamboa et al., 2013). CENPK expression is shown for each risk group (Fig. 3B) which exhibited significant differences (*p* = 0.0126) in clinical outcomes according to the Kaplan–Meier survival analysis (Fig. 3C).

### CENPK improves the sensitivity of clinical outcomes of CA125 or HE4 for patients with ovarian cancer

**Figure 4:**
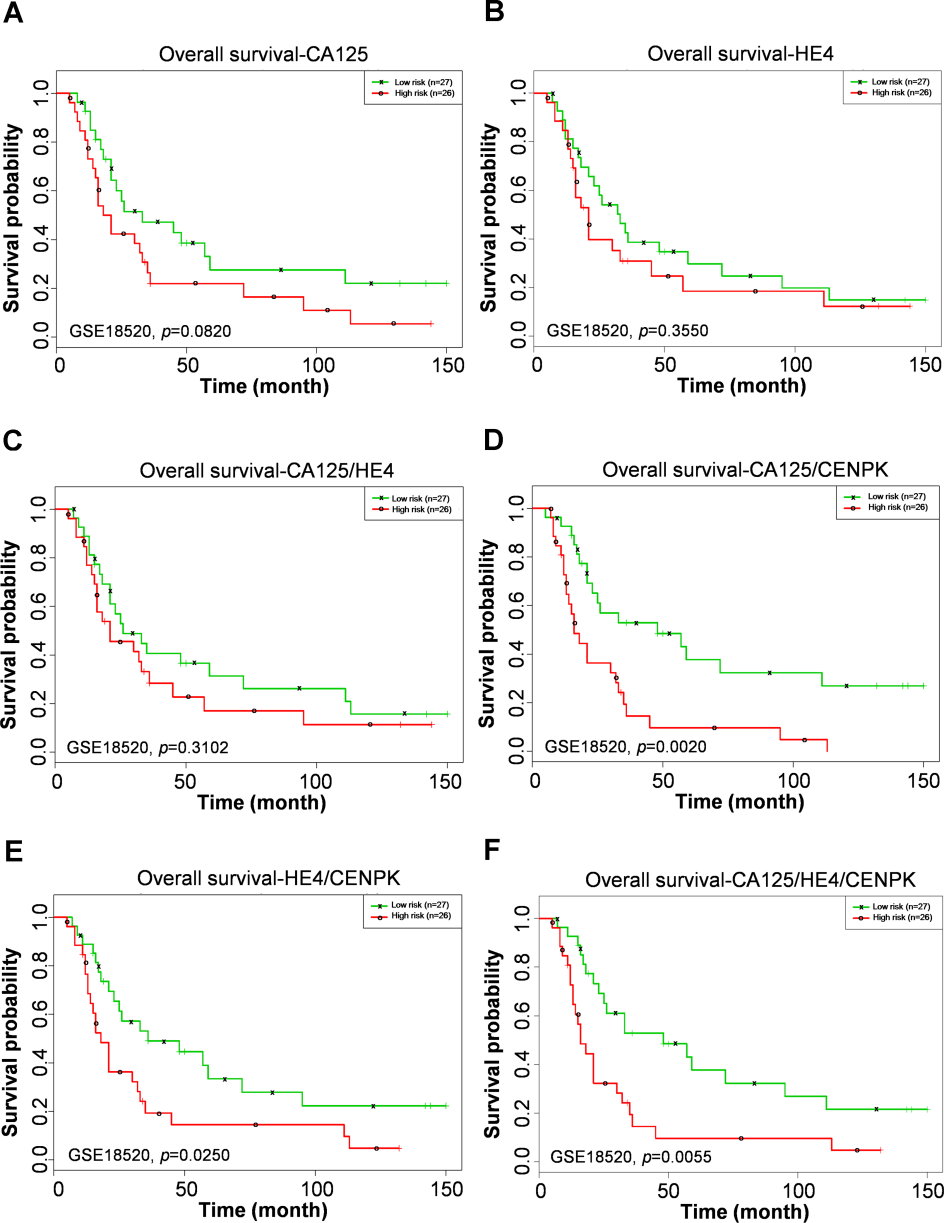
Kaplan–Meier curves according to various combinations of single-gene, two-gene, and three-gene models. Clinical outcomes for the single genes of CA125 (A) or HE4 (B) or combinations of CA125/HE4 (C), CA125/CENPK (D), HE4/CENPK (E), and CA125/HE4/CENPK (F) mRNA status of ovarian cancer patients.

Cancer antigen 125 (CA125) and human epididymis protein 4 (HE4) are the gold-standard tumor markers for ovarian cancer. To understand whether there is a correlation between CA125 levels and survival rates of ovarian cancer patients, the above-mentioned cohort (GSE18520) of 53 ovarian cancer patients was analyzed using a Kaplan–Meier survival analysis. As shown in Fig. 4A, there was no significant difference in expression levels of CA125 according to the Kaplan–Meier survival analysis. Next, the sensitivity of HE4 for predicting ovarian cancer survival was evaluated. As shown in Fig. 4B, expression levels of HE4 mRNA were not significantly associated with the clinical outcomes of ovarian cancer patients. Moreover, there was also no significant difference in CA125 and HE4 two-gene models according to the Kaplan–Meier survival analysis (Fig. 4C). Taken together, these results indicate that correlations of survival with CA125, HE4, and the combination of CA125 and HE4 mRNA expressions were not associated with poor prognoses in ovarian cancer patients. Further, to understand whether CENPK can complement CA125 or HE4 to improve the sensitivity of clinical outcomes of ovarian cancer patients, combinations of two- and three-gene models were analyzed using the Kaplan–Meier survival analysis. Specifically, as shown in Figs. 4D–4F, significant differences in genes selected by any combination of the two- or three-gene models in clinical outcomes were exhibited according to the Kaplan–Meier survival analysis; in particular, the most significant model was the combination of CA125 and CENPK mRNA expressions which was associated with poor prognoses in ovarian cancer patients (*p* = 0.0020).

## Discussion

Ovarian cancer remains the deadliest gynecologic malignancy in the advanced stage in developed countries. Approximately two-thirds of new cases are diagnosed when metastases are already beyond the pelvis, which explains the low 5-year survival rate of 27% (Siegel et al., 2014). Thus, the high sensitivity of tumor markers for early detection of ovarian cancer is the most essential determinant of survival. In this study for the first time, we identified the important role played by a member of the CENP family proteins, CENPK, as a novel tumor marker of ovarian cancer. There are several lines of evidence that support this conclusion. First, among CENP family proteins, CENPK was specifically upregulated in ovarian cancer tissues and cell lines. Second, RNAi-mediated CENPK knockdown caused a significant decrease in the growth rate of ovarian cancer cells, which implies that CENPK has an oncogenic role. Third, high expression of CENPK by clinical ovarian tumors was associated with a shorter survival of ovarian cancer patients. Fourth, CENPK can complement CA125 or HE4 to significantly improve the sensitivity of clinical outcomes of ovarian cancer patients. Collectively, this study is the first to report that CENPK is upregulated in ovarian cancer tissues and cell lines and also showed that high CENPK expression in ovarian tumors is a strong predictor of a poor prognosis.

The best-studied and longest-utilized biochemical marker of epithelial ovarian cancer is CA125 (Cohen et al., 2014), an antigenic determinant on a high-molecular-weight glycoprotein found on the epithelial surface of reproductive tract organs and the peritoneum and recognized by the murine monoclonal antibody, OC-125 (Yin & Lloyd, 2001). The Scientific Societies Clinical Guidelines recommends CA125 as a tumor marker for detecting recurrence, monitoring therapy, and determining the prognosis of women with ovarian cancer (Tang et al., 2008). However, although CA125 is overexpressed in 80% of epithelial ovarian cancer cases and is the tumor marker of choice for epithelial ovarian cancer (Bast, Hennessy & Mills, 2009), this marker naturally increases with ovulation and may be elevated with endometriosis, fibroids, and many other benign conditions (Etzioni et al., 2003). In addition, the effect of the differential CA125 gene expression on patient survival is still controversial (Kobayashi et al., 2012). In our study, we found that there was no correlation between the expression levels of CA125 and clinical outcomes of ovarian cancer patients according to the Kaplan–Meier survival analysis. This result also corresponds with previous findings (Ortiz-Munoz et al., 2014) and indicates that CA125 alone is not recommended for predicting ovarian cancer survival. Currently, HE4, also known as whey acidic protein four disulfide core 2 (WFDC2), is the only biomarker, other than CA125, which has been approved by the US Food and Drug Administration as a diagnostic marker for ovarian cancer (Hellstrom et al., 2003; Simmons, Baggerly & Bast, 2013). Measurement of HE4 in serum is a new approach for diagnosing ovarian cancer. The reproductive tract, respiratory tissues, and salivary glands are the main sources expressing HE4, it also is remarkably increased by ovarian cancer cells, and it is considered one of the main tumor markers, especially in specific types of ovarian cancer including serous or endometrial carcinoma (Galgano, Hampton & Frierson, 2006; Nagy et al., 2012). Previous studies showed that HE4 has a similar sensitivity to that of CA125 but has an increased specifically in patients with malignant gynecological diseases compared to those with benign gynecological diseases (Escudero et al., 2011; Nolen et al., 2010). Moreover, it was also reported that HE4 has a better capacity than CA125 to distinguish among healthy women and women with benign disease from those with malignant tumors (Moore et al., 2009; Park et al., 2011). Nevertheless, HE4 is still not specific for ovarian cancer due to abnormal levels found in other malignancies such as lung cancer and endometrial adenocarcinomas (Escudero et al., 2011; Galgano, Hampton & Frierson, 2006; Moore et al., 2008). Collectively, in the present study, we show that the presence of CENPK can significantly improve the sensitivity of CA125 or HE4 for predicting clinical outcomes of ovarian cancer patients.

Chromosomal aberrations are a cardinal feature of carcinogenesis, and identifying amplified or deleted chromosomal regions associated with cancer would elucidate the underlying pathogenetic mechanisms (Davare & Tognon, 2015). Comparative genomic hybridization (CGH) is a molecular cytogenetic method that detects global DNA sequence copy number changes in tumor genomes (Kallioniemi et al., 1994). CGH has extensively been applied to analyze genomic changes in ovarian cancer (Helou et al., 2006). Micci et al. (2014) studied cytogenetic aberrations of ovarian carcinoma by karyotyping and high-resolution CGH. They found that over 60% of clear cell ovarian cancer with multiple areas of chromosomal gain were often scored on 1q41-44, 2p13, 2p22-23, 2q12-13, 2q23-32, 3q13-24, 5q12-23, 5q32-34, 7p13, 7q21-34, 8q11, 10q11, 10q23-25, 12p11-13, 17q22-23, 19q13, 20q, and 22q11-12. Many oncogenes within these regions were demonstrated to be associated with ovarian cancer; for example, Akt3 (located at 1q44) (Cristiano et al., 2006), hepatocyte growth factor (HGF) (located at 7q21), and its receptor, MET (located at 7q34) (Tang et al., 2010), KRAS (located at 12p12) (Ratner et al., 2012), and AURKA (located at 20q) (Gritsko et al., 2003) were found to frequently be overexpressed in ovarian cancer. CENPK was reported as located on chromosome 5q12.3 and belonged to the chromosome region of 5q12-23 (Gerhard et al., 2004). In addition, amplification of chromosome 5q11-14 has been reported as associated with poor survival of ovarian cancer patients (Thomassen et al., 2009). However, no oncogenic genes have been identified in the amplified region of chromosome 5q11-14 as contributing to poor outcomes of ovarian cancer. In this study, we revealed for the first time that CENPK was overexpressed in ovarian cancer cell lines and tissues and its overexpression was associated with poor outcomes of ovarian cancer. Thus, we conclude that CENPK is a novel oncogene of ovarian cancer and located on amplified region of chromosome 5q11-14 in ovarian cancer.

Carcinogenesis occurs when kinetochores become functionally unstable, leading to abnormal segregation of chromosomes and consequent genetic instability (Goncalves Dos Santos Silva et al., 2008; Kops, Weaver & Cleveland, 2005). CENPA was the first centromeric protein identified, and it was reported that CENPA overexpression can potentially lead to the spread of centromere heterochromatin along chromosome arms causing defects in microtubule-kinetochore anchoring and eventually causing genomic instability (Amato et al., 2009). To date, many reports have shown a link between CENPA and various human cancers (Li et al., 2011; McGovern et al., 2012; Tomonaga et al., 2003; Wu et al., 2012), including ovarian cancer (Qiu et al., 2013). Overexpression of CENPA was demonstrated to be associated with poor clinical outcomes of ovarian cancer patients (Qiu et al., 2013). In this study, however, we found that the presence of CENPA did not increase the sensitivity of CA125 or HE4 for predicting ovarian cancer outcomes (compare Figs. S1A and S1B with Figs. 4A and 4B). Collectively, although CENPA is an essential factor in kinetochore assembly and its overexpression is associated with high growth activity of cancer cells, the combination of CENPK and CA125 or HE4 was a more accurate predictor than the combination of CENPA and CA125 or HE4 in the prognosis of ovarian cancer.

## Conclusions

Our study showed for the first time the important role played by CENPK. We identified that CENPK is specifically upregulated in ovarian cancer cells, and its overexpression is associated with a poor prognosis in patients with ovarian cancer. Moreover, incorporating CENPK with the gold standard tumor markers, CA125 or HE4, can improve the sensitivity of CA125 or HE4 for predicting ovarian cancer outcomes.

## Supplemental Information

10.7717/peerj.1386/supp-1 Data S1 Raw data and Fig. S1

10.7717/peerj.1386/supp-2 Figure S1 Kaplan–Meier curves according to combinations of two-gene models Overall survival for combinations of CA125/CENPA (A) and HE4/CENPA (B) mRNA status of ovarian cancer patients.
