# Supplementary material for: Overexpression of centromere protein K (CENPK) in ovarian cancer is correlated with poor patient survival and associated with predictive and prognostic relevance
Source: PeerJ. 2015 Nov 5;3:e1386. doi: 10.7717/peerj.1386 (PMC4647587; doi:10.7717/peerj.1386)
Supplement: Data S1 [file peerj-03-1386-s001.zip › Raw data/Supplemental Fig. S1.docx]

**Supplemental Information**

**Title: Overexpression of centromere protein K (CENPK) in ovarian cancer is correlated with poor patient survival and associated with predictive and prognostic relevance**

Authors: Yi-Chao Lee, Chi-Chen Huang, Ding-Yen Lin, Wen-Chang Chang, Kuen-Haur Lee

Supplemental Information includes 1 Supplemental Figure


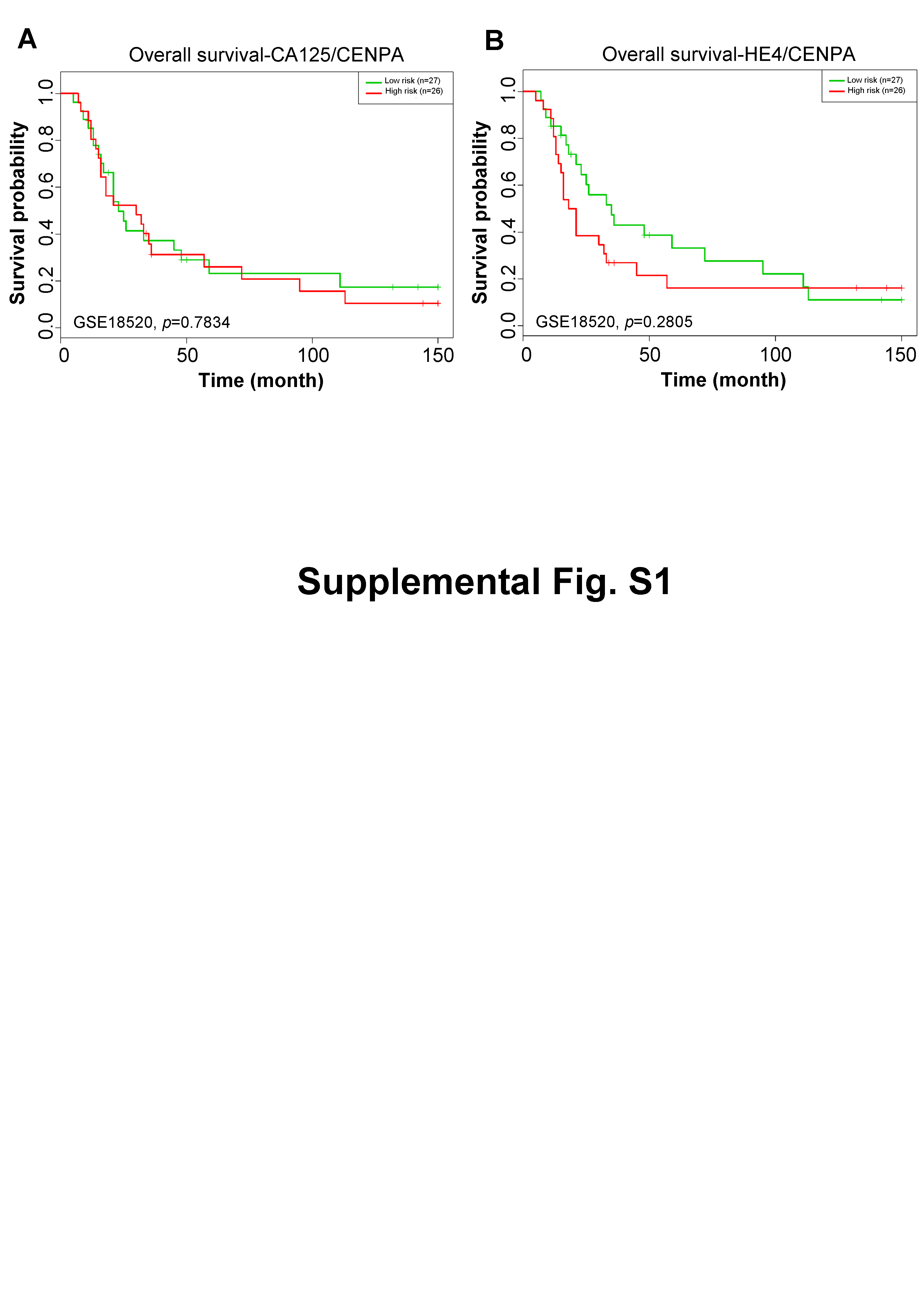


**Fig. S1 Kaplan-Meier curves according to combinations of two-gene models.** Overall survival for combinations of CA125/CENPA (A) and HE4/CENPA (B) mRNA status of ovarian cancer patients.
