# Supplementary figures and images for: Overexpression of centromere protein K (CENPK) in ovarian cancer is correlated with poor patient survival and associated with predictive and prognostic relevance
Source: PeerJ. 2015 Nov 5;3:e1386. doi: 10.7717/peerj.1386 (PMC4647587; doi:10.7717/peerj.1386)

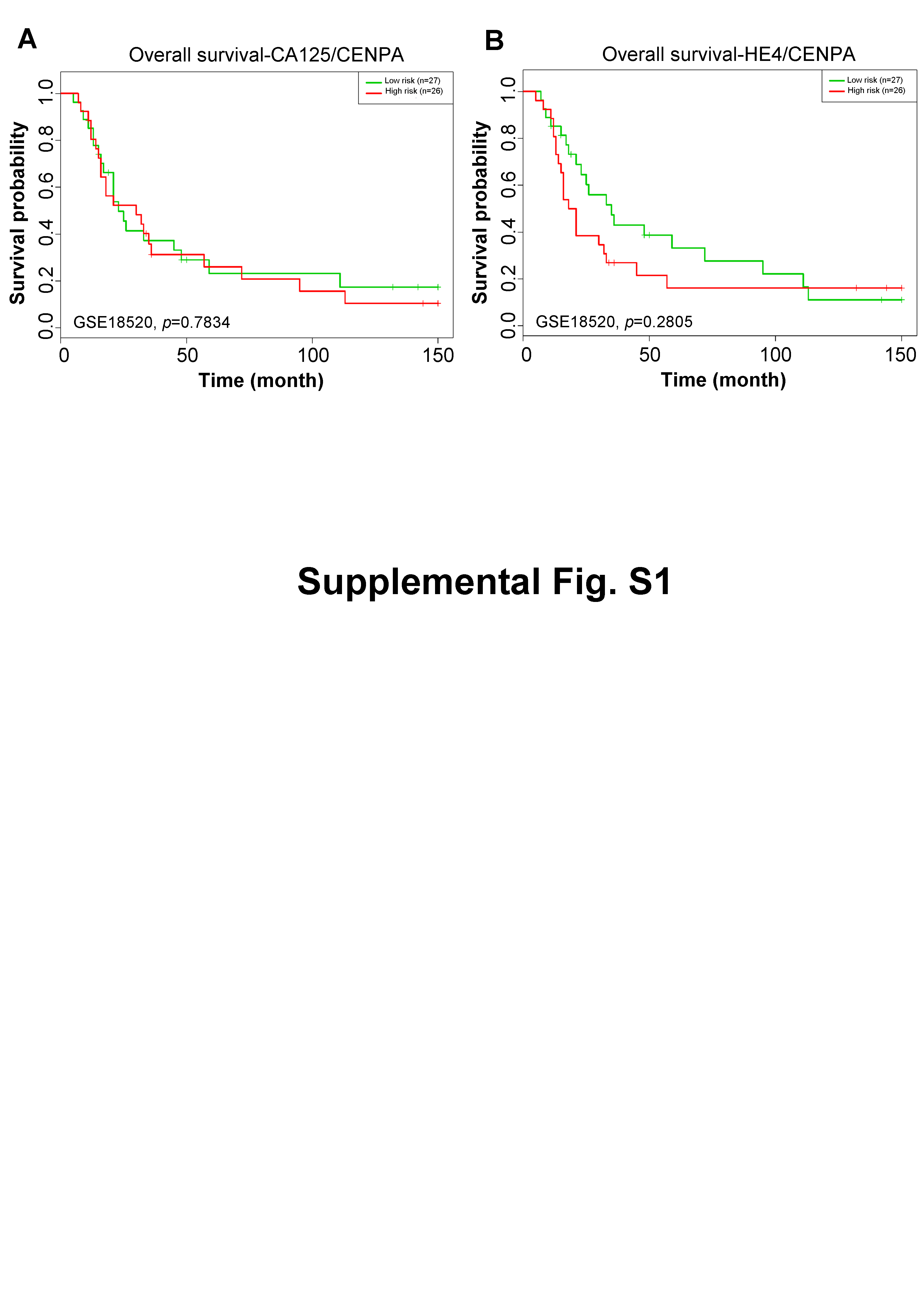

Supplement: Figure S1 — Overall survival for combinations of CA125/CENPA (A) and HE4/CENPA (B) mRNA status of ovarian cancer patients. [file peerj-03-1386-s002.png]
